# Correction to: Metabolic reprogramming of T regulatory cells in the hypoxic tumor microenvironment

**DOI:** 10.1007/s00262-021-02999-0

**Published:** 2021-07-07

**Authors:** Varun Sasidharan Nair, Reem Saleh, Salman M. Toor, Farhan S. Cyprian, Eyad Elkord

**Affiliations:** 1 Cancer Research Center, Qatar Biomedical Research Institute (QBRI), Hamad Bin Khalifa University (HBKU), Qatar Foundation (QF), Doha, Qatar; 2 Department of Basic Medical Sciences, College of Medicine, Member of QU Health, Qatar University, Doha, Qatar; 3 Biomedical Research Center, School of Science, Engineering and Environment, University of Salford, Manchester, M5 4WT UK

## Correction to: Cancer Immunology, Immunotherapy 10.1007/s00262-020-02842-y

The article Metabolic reprogramming of T regulatory cells in the hypoxic tumor microenvironment, written by Varun Sasidharan Nair, Reem Saleh, Salman M. Toor, Farhan S. Cyprian and Eyad Elkord, was originally published electronically on the publisher’s internet portal on 03 February 2021 without open access. With the author(s)’ decision to opt for Open Choice the copyright of the article changed on 25 May 2021 to © The Author(s) 2021 and this article is licensed under a Creative Commons Attribution 4.0 International License, which permits use, sharing, adaptation, distribution and reproduction in any medium or format, as long as you give appropriate credit to the original author(s) and the source, provide a link to the Creative Commons licence, and indicate if changes were made. The images or other third party material in this article are included in the article’s Creative Commons licence, unless indicated otherwise in a credit line to the material. If material is not included in the article’s Creative Commons licence and your intended use is not permitted by statutory regulation or exceeds the permitted use, you will need to obtain permission directly from the copyright holder. To view a copy of this licence, visit http://creativecommons.org/licenses/by/4.0/.

The original article has been corrected.

